# Supplementary material for: Developmental Ethanol Exposure Impacts Purkinje Cells but Not Microglia in the Young Adult Cerebellum
Source: Cells. 2024 Feb 23;13(5):386. doi: 10.3390/cells13050386 (PMC10930603; doi:10.3390/cells13050386)
Supplement: Supplementary file 1 [file cells-13-00386-s001.zip › cells-2876612-supplementary.pdf]

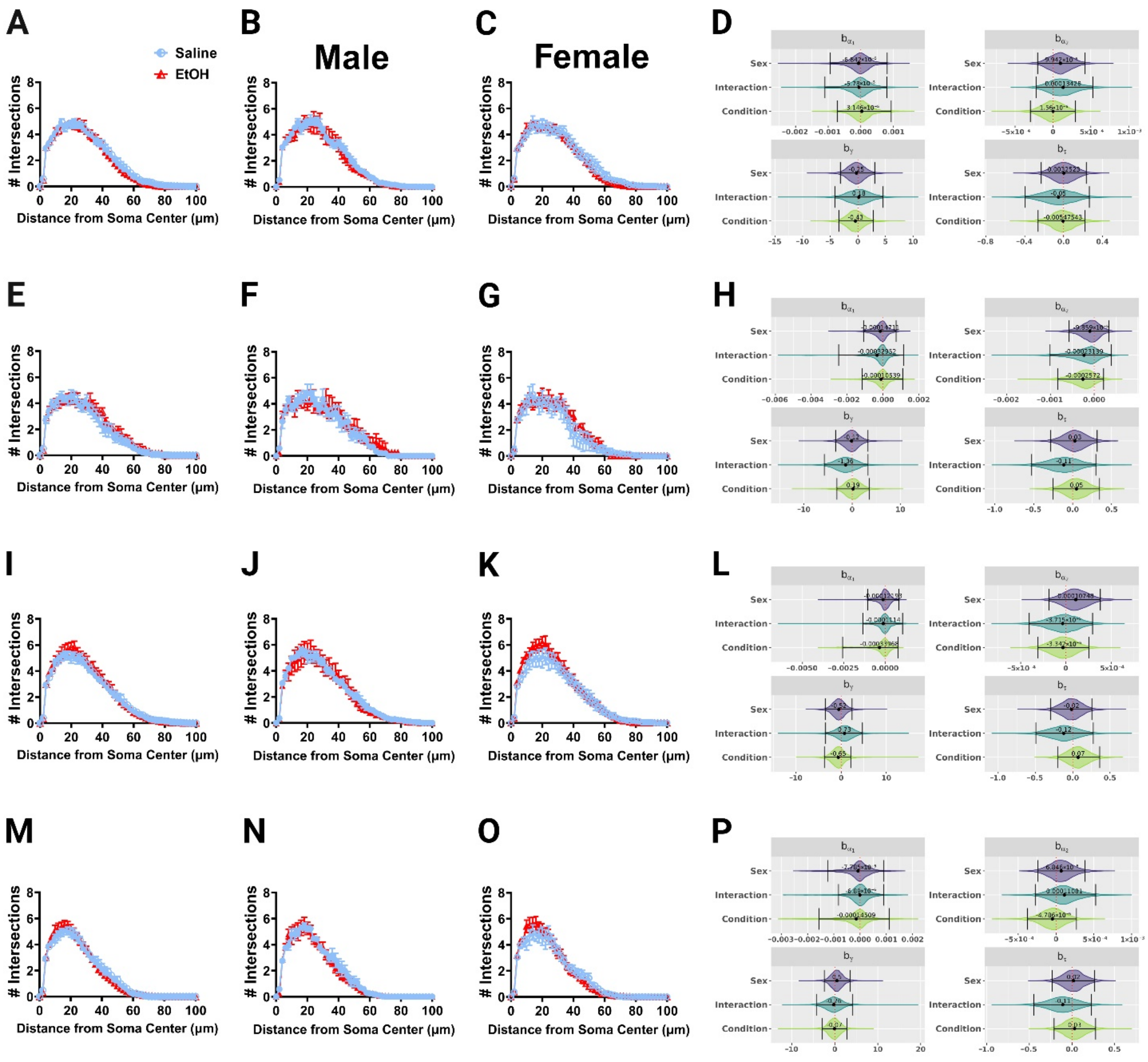

Figure S1: Cerebellar microglia morphology in each layer of lobule IV/V in fixed tissue. (**A-C, E-G, I-K, M-O)** Sholl curves for the ML (**A-C**), PCL (**E-G**), GL (**I-K**), and WM (**M-O**). For each layer, sexes were combined (**A, E, I, M**) or separated into males (**B, F, J, N**) and females (**C, G, K, O**). There were no differences in ramification due to treatment or sex in any layer. (**D, H, L, P**) Individual Sholl curves were fit using a hierarchical Bayesian approach to capture variation at each level of the experimental hierarchy. 95% credible intervals for effects on each parameter from (**Figure 3B**) were calculated across treatments and sexes in the ML (**D**), PCL (**H**), GL (**L**), and WM (**P**). **(A-L)** Each datapoint represents a treatment group and sex. Data are presented as the mean ± SEM.

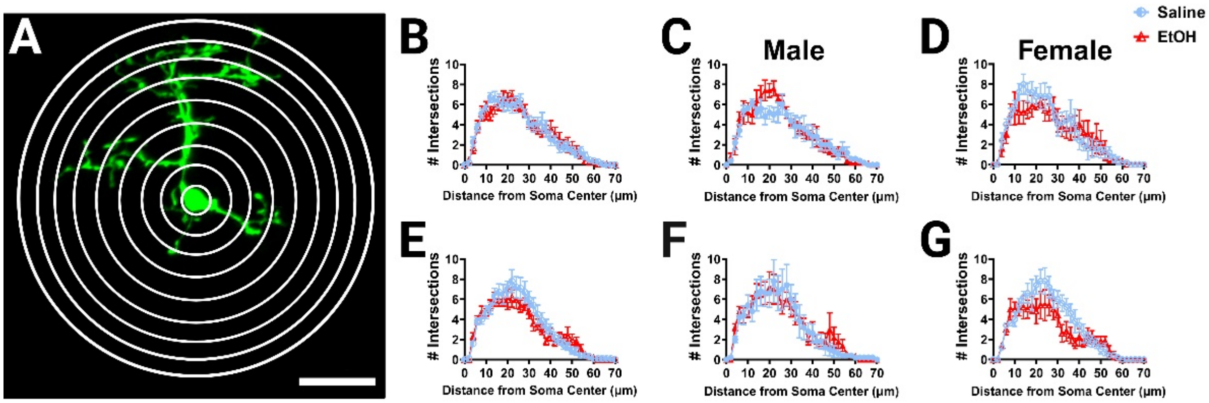

Figure S2: Cerebellar microglia morphology *in vivo*. (A) *In vivo* two-photon image of cerebellar microglia. Concentric rings were drawn around each microglia to examine process ramification. **(B-G)** Sholl curves for microglia in the ML (**B-D)** and PCL (**E-G)** when sexes were combined (**B, E**) or separated into males (**C, F**) or females (**D,G**). **(B-G)** Each datapoint represents a treatment group and sex. Data are presented as the mean ± SEM. Scale bar= 25µm

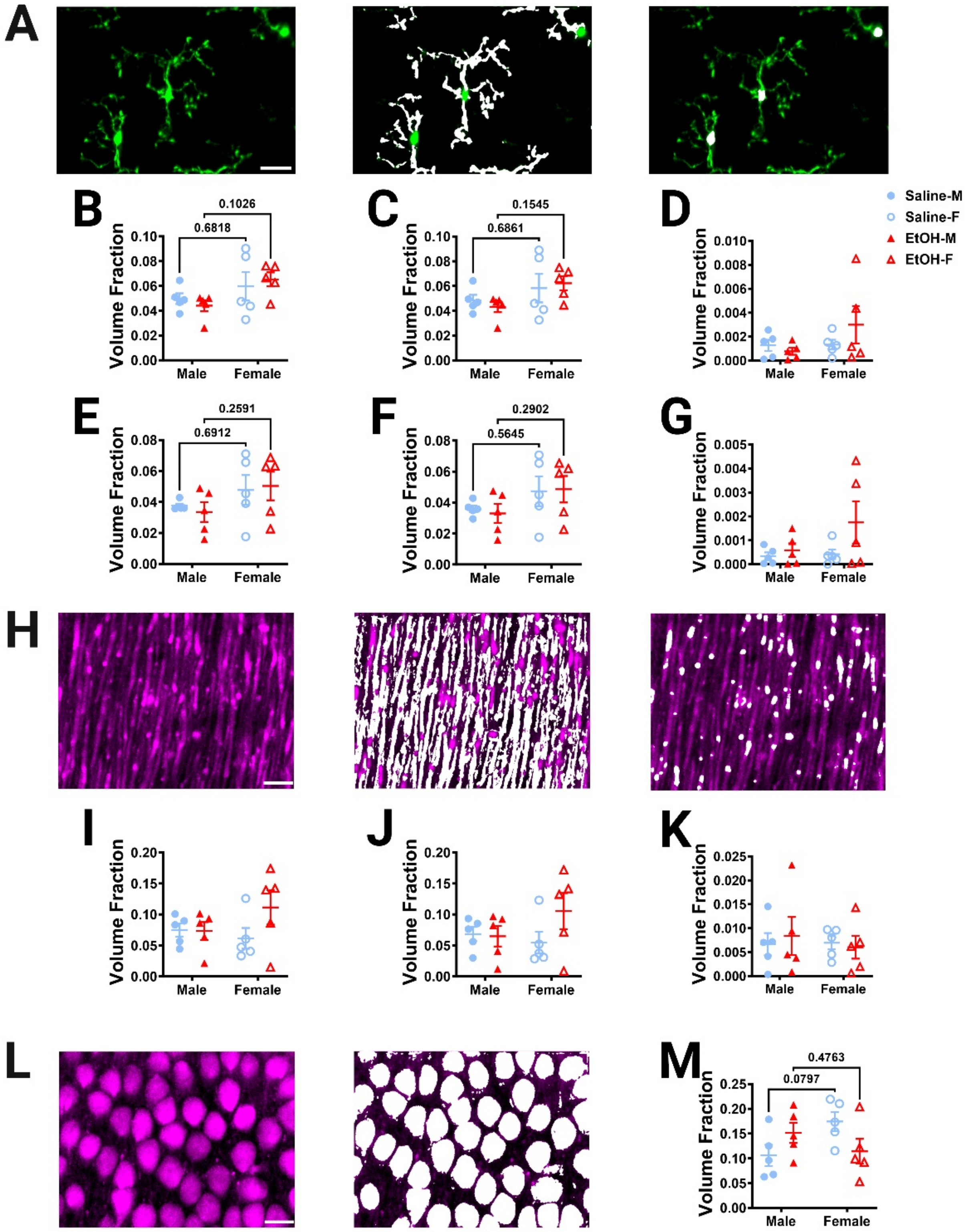

Figure S3. Cerebellar microglia and Purkinje cell subcomponents *in vivo*. (A, H, L) *In vivo* two-photon images showing microglia (**A**), Purkinje cell dendrites and branch points in the ML (**H**), and Purkinje cell somas in the PCL (**L**). White overlay indicates subcomponents for each cell type. **(B-G)** Whole microglia (**B, E**) were divided into processes (**C, F**) and somas (**D, G**) in either the ML (**B-D**) or PCL (**E-G**). (**B**) ML whole microglia pixel numbers had no main effect for treatment (F (1, 16) = 5.406e-006, p= 0.9982) and no

treatment-sex interaction effect (F (1, 16) = 0.6330, p= 0.4379), but female mice had significantly more microglia pixels than male mice (F (1, 16) = 4.769, p= 0.0442), with a trend in the ethanol-dosed group (p= 0.1026). (**C**) ML microglia process pixel numbers had no main effect for treatment (F (1, 16) = 0.007151, p= 0.9337) and no treatment-sex interaction effect (F (1, 16) = 0.4152, p= 0.5285), but female mice had a trend towards more microglia pixels than male mice (F (1, 16) = 4.106, p= 0.0597). (**D**) ML microglia soma pixel numbers had no main effect for treatment (F (1, 16) = 0.4740, p= 0.5010), or sex (F (1, 16) = 1.790, p= 0.1996), and no treatment-sex interaction effect (F (1, 16) = 1.621, p= 0.2211). (**E**) PCL whole microglia pixel numbers had no main effect for treatment (F (1, 16) = 0.009064, p= 0.9253) and no treatment-sex interaction effect (F (1, 16) = 0.1963, p= 0.6637), but female mice had a trend towards more microglia pixels than male mice (F (1, 16) = 3.303, p= 0.0879). (**F**) PCL microglia process pixel numbers had no main effect for treatment (F (1, 16) = 0.01486, p= 0.9045) and no treatment-sex interaction effect (F (1, 16) = 0.08783, p= 0.7708), but female mice had a trend towards more microglia pixels than male mice (F (1, 16) = 3.497, p= 0.0799). (**G**) PCL microglia soma pixel numbers had no main effect for treatment (F (1, 16) = 2.652, p= 0.1230), or sex (F (1, 16) = 1.644, p= 0.2180), and no treatment-sex interaction effect (F (1, 16) = 1.254, p= 0.2793). **(I-K)** Whole Purkinje cells in the ML (**I**) were divided into dendrites (**J**) and branchpoints (**K**). (**I**) ML whole Purkinje cell pixel numbers had no main effect for treatment (F (1, 16) = 1.695, p= 0.2113), or sex (F (1, 16) = 0.4414, p= 0.5159), and no treatment-sex interaction effect (F (1, 16) = 1.938, p= 0.1829). (**J**) ML Purkinje cell dendrite pixel numbers had no main effect for treatment (F (1, 16) = 1.445, p= 0.2467), or sex (F (1, 16) = 0.4560, p= 0.5091), and no treatment-sex interaction effect (F (1, 16) = 1.887, p= 0.1884). (**K**) ML Purkinje cell branch pixel numbers had no main effect for treatment (F (1, 16) = 0.02213, p= 0.8836), or sex (F (1, 16) = 0.1447, p= 0.7087), and no treatment-sex interaction effect (F (1, 16) = 0.2422, p= 0.6293). **(M)** Purkinje cell somas were identified in the PCL. PCL Purkinje cell soma pixel numbers had no main effect for treatment (F (1, 16) = 0.1150, p= 0.7389), or sex (F (1, 16) = 0.5121, p= 0.4845), but there was a significant treatment-sex interaction effect (F (1, 16) = 5.996, p= 0.0262) with a post hoc trend of saline females having more pixels than saline males (p=0.0797). (**B-G, I-K, M**) Each datapoint represents an individual animal. Data are presented as the mean ± SEM. Two-way ANOVA with Bonferroni post hoc comparisons. Scale bar= 25μm

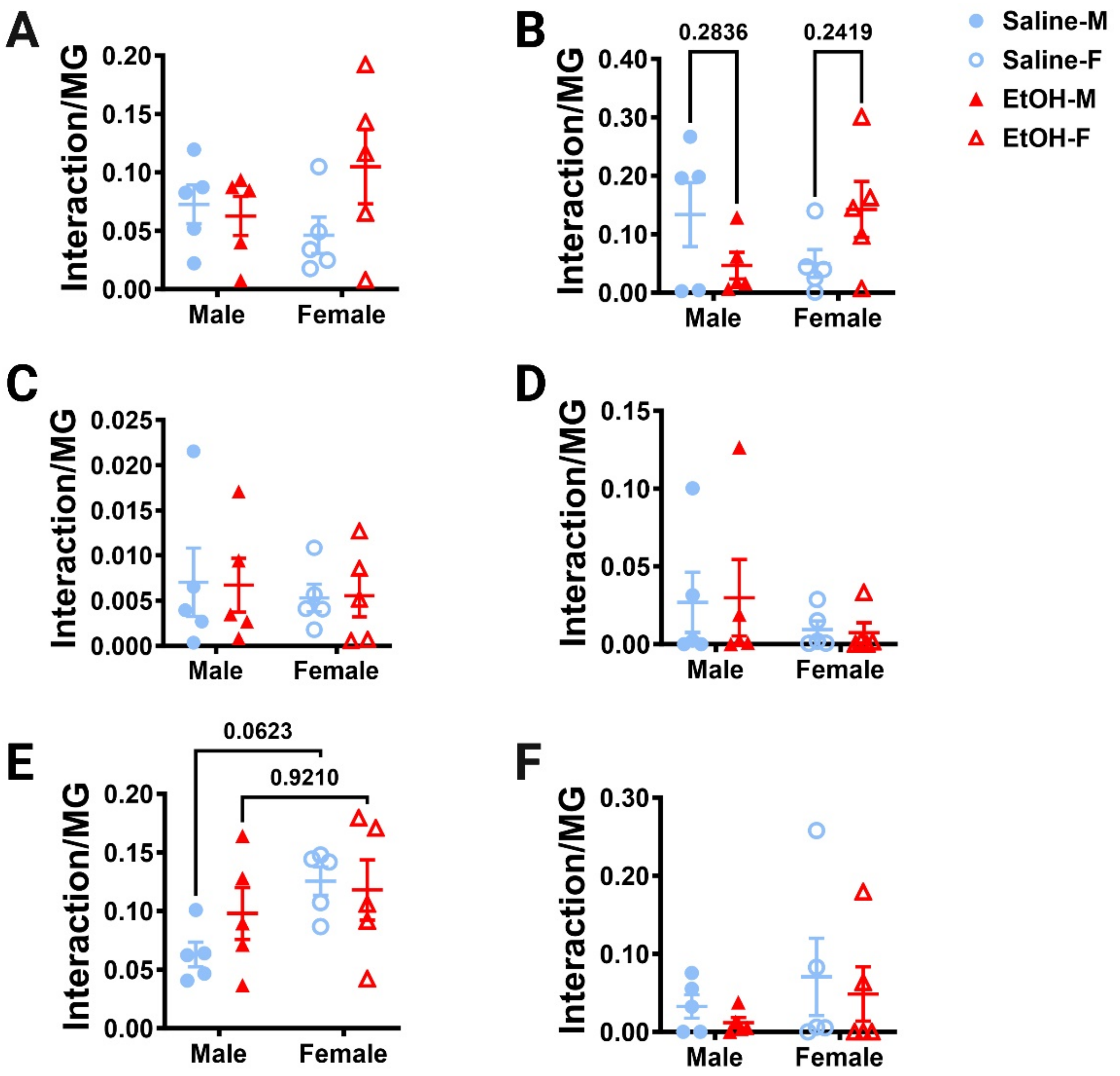

Figure S4. Cerebellar microglia-Purkinje cell subcomponent interactions *in vivo*. (A-F) The overlap between the microglia and Purkinje cell subcomponent pixels were normalized to the number of the microglia pixels to determine microglia-Purkinje cell interaction in the ML (**A-D**) and PCL (**E-F**). (**A**) ML microglia process x Purkinje cell dendrite interactions had no main effect for treatment (F (1, 16) = 1.317, p= 0.2679), or sex (F (1, 16) = 0.1365, p= 0.7167), and no treatment-sex interaction effect (F (1, 16) = 2.615, p= 0.1254). (**B**) ML microglia soma x Purkinje cell dendrite interactions had no main effect for treatment (F (1, 16) = 0.004292, p= 0.9486), or sex (F (1, 16) = 0.02414, p= 0.8785), but there was a significant treatment-sex interaction effect (F (1, 16) = 5.067, p= 0.0388). (**C**) Microglia process x Purkinje cell branch interactions had no main effect for treatment (F (1, 16) = 0.0001596, p= 0.9901), or sex (F (1, 16) = 0.2689, p= 0.6112), and no treatment-sex interaction effect (F (1, 16) = 0.009910, p= 0.9219). (**D**) ML microglia soma x Purkinje cell branch interactions had no main effect for treatment (F (1, 16) = 0.0009207, p= 0.9762), or sex (F (1, 16) = 1.521, p= 0.2352), and no treatment-sex interaction effect (F (1, 16) = 0.02347, p= 0.8802). (**E**) PCL microglia process x Purkinje cell soma interactions had no main effect for treatment (F (1, 16) = 0.5339, p= 0.4756) and no treatment-sex interaction effect (F (1, 16) = 1.290, p= 0.2727), but female mice had significantly more interactions than male mice (F (1, 16) = 4.863, p= 0.0424), with a trend apparent in saline-dosed mice

(p=0.0623). (**F**) PCL microglia soma x Purkinje cell soma interactions had no main effect for treatment (F (1, 16) = 0.4601, p= 0.5073), or sex (F (1, 16) = 1.418, p= 0.2511), and no treatment-sex interaction effect (F (1, 16) = 0.0003324, p= 0.9857). **(A-F)** Each datapoint represents an individual animal. Data are presented as the mean ± SEM. Two-way ANOVA with Bonferroni post hoc comparisons.
